# Surgeon-Performed Ultrasound in Diagnosing Acute Cholecystitis and Appendicitis

**DOI:** 10.1007/s00268-018-4673-z

**Published:** 2018-06-07

**Authors:** Camilla Gustafsson, Anna Lindelius, Staffan Törngren, Hans Järnbert-Pettersson, Anders Sondén

**Affiliations:** 1 Department of Clinical Science and Education, Department of Surgery, Karolinska Institutet, Södersjukhuset (Stockholm South General Hospital), Sjukhusbacken 10, 118 83 Stockholm, Sweden; 2 Saltsjö-Boo, Sweden; 3 Department of Clinical Science and Education, Karolinska Institutet, Södersjukhuset, Stockholm, Sweden

## Abstract

**Background:**

The use of ultrasound (US) outside the radiology department has increased the last decades, but large studies assessing the quality of bedside US are still lacking. This study evaluates surgeon-performed US (SPUS) and radiologist-performed US (RPUS) with respect to biliary disease and appendicitis.

**Methods:**

Between October 2011 and November 2012, 300 adult patients, with a referral for an abdominal US, were prospectively enrolled in the study and examined by a radiologist as well as a surgeon. The surgeons had undergone a 4-week-long US education. US findings of the surgeon and of the radiologist were compared to final diagnosis, set by an independent external observer going through each patient’s chart.

**Results:**

Among 183 patients with suspected biliary disease, 74 had gallstones and 21 had acute cholecystitis. SPUS and RPUS diagnosed gallstones with a sensitivity of 87.1 versus 97.3%. Specificity was 96.0 versus 98.9%, and the accuracy 92.3 versus 98.2%. The sensitivity, specificity and accuracy for acute cholecystitis by SPUS and RPUS were: 60.0 versus 80.0%, 98.6 versus 97.8% and 93.9 versus 95.6%, respectively. Among 58 patients with suspected appendicitis, 15 had the disease. The sensitivity, specificity and accuracy for appendicitis by SPUS and RPUS were: 53.3 versus 73.3%, 89.7 versus 93.3% and 77.3 versus 86.7%, respectively.

**Conclusion:**

SPUS is reliable in diagnosing gallstones. Diagnosing cholecystitis and appendicitis with US is more challenging for both surgeons and radiologists.

**Trial registration number:**

The study was registered at clinicaltrials.gov. Registration number: NCT02469935.

## Introduction

The use of ultrasound (US) outside the radiology department, often referred to as point-of-care ultrasound (POCUS), has increased in the last decades as more compact and portable scanners have become available [1]. At Stockholm South General Hospital’s surgery department, abdominal POCUS has been part of surgical resident training since 2004. We have previously shown that surgeon-performed ultrasound (SPUS) at the emergency department (ED) results in fewer additional examinations, fewer admissions and shorter lead times to surgery [2].

We, and others, have demonstrated that SPUS can detect gallstones with high diagnostic accuracy [3–7]. Using the same patient cohort as in our recent study [7], our current work focuses on the diagnosis of cholecystitis and appendicitis, two common causes of acute abdominal pain [8]. Previous work on the diagnostic accuracy of radiologist-performed ultrasound (RPUS)—in cholecystitis and appendicitis—shows variable results. The reported sensitivity differs, ranging from 50 to 100% for cholecystitis [8, 9] and 52–76% for appendicitis [8, 10]. The quality of abdominal US—in these contexts—appears to be even more operator dependent, which may have negative impact on the quality of SPUS since surgeons don’t get the same amount of US training as radiologists [11]. To what extent this matters, however, is not known, since studies on the subject are few [12]. The aim of this study was to validate the diagnostic accuracy of SPUS regarding acute cholecystitis and appendicitis, comparing ultrasound examinations to final diagnosis. For comparison, we examined the accuracy of RPUS using the same reference standard. To estimate the overall US competence of the participating radiologists and surgeons, we also included the diagnostic accuracy of detecting gallstones in the analysis.

## Materials and methods

### Enrollment of patients

Three hundred patients, referred to the radiology department at Stockholm South General Hospital, for any diagnostic abdominal US examination, were enrolled between October 2011 and November 2012, and informed consent was obtained. Exclusion criteria were age <18 years, inability to communicate with the examiner and referrals concerning metastases of the liver or contrast-enhanced examinations.

### Data collection

Enrolled patients received one US examination by the study surgeon as well as the standard US examination by the on-duty radiologist. The examining surgeon and radiologist were blinded to each other’s findings, and examinations were done right after one another when possible, and always within 6 h from each other. The surgeon took a short history from the patient and then performed the US, following a standardized protocol. Each examination took the surgeon approximately 15 min (10–20) to perform. The on-duty radiologist performed a standard care US focusing on the individual referrals, and each examination took approximately 10 min (5–15). Among the radiologists, the major part of the scans was done by US-specialized radiologists with several years of training (73% of the scans were performed by specialists in radiology and the remaining 27% by radiologists in specialist training). The surgeons used a portable US machine of the model LOGIQ e with a convex (1.6–4.6 MHz) or linear (5–13 MHz) transducer, GE Healthcare, WuXi, China. The radiologists used Philips iU22 with a convex C5-1 or a linear L12-5 transducer.

### Criteria for patient inclusion

Patients with suspected biliary disease and/or suspected appendicitis were considered eligible for inclusion. Suspected biliary disease was defined as patients presenting with pain in the right upper quadrant (RUQ) and/or tenderness in the RUQ during physical examination and/or with a referral to the radiology department regarding gallstones and/or cholecystitis.

Suspected appendicitis was defined as patients presenting with pain in the right lower quadrant and/or tenderness in the right lower quadrant and/or with a referral to the radiology department regarding appendicitis.

### Reference standard

The final diagnosis was set by an independent external observer, a senior consultant surgeon, based on discharge diagnosis, operation logs and pathology reports from each patient’s chart. Findings of gallstones, acute cholecystitis and/or appendicitis were marked with “Yes” or “No” for each patient and each diagnosis in a separate protocol. The diagnosis of acute cholecystitis was set using the Tokyo Guidelines 2013 (TG13) criteria [13] together with operation logs and pathology reports.

### US training of participating surgeons

Six study surgeons, five residents in their final years and one specialist in surgery, attended a 1-week course on US physics, technique, anatomy and hands-on training, led by specialists in US, followed by 3 weeks of training in the radiology department. The training has been thoroughly described previously [7]. After completing the training, each surgeon spent 2 weeks enrolling and scanning patients during office hours in the hospital’s radiology department.

### Ethics

The Ethical Review Board at Karolinska Institutet, Stockholm, Sweden, approved the study (2011/1025-31/1).

### Statistical analysis

We calculated sensitivity, specificity, overall accuracy, positive predicted value (PPV), negative predicted value (NPV), positive likelihood ratio (LR+) and negative likelihood ratio (LR−) for SPUS and RPUS in detecting gallstones, cholecystitis and appendicitis, respectively. Final diagnosis, defined above, was set as reference standard. We calculated the inter-observer agreement between surgeons and radiologists for each of the three diagnoses using Cohen’s kappa. The sample size of 300 patients comes from a power calculation in a previous study designed to detect a difference between SPUS and RPUS in detecting gallstones [7]. We used the same cohort for these additional diagnoses. To study if there was any systematic difference between how often the surgeon and the radiologist set each diagnosis, we used McNemar’s test. A *p* value <0.05 (two tailed) was considered statistically significant. Analyses were done in IBM SPSS Statistics, version 23. We used the efficient score due to Wilson to calculate the 95% confidence intervals (CI) of sensitivity, specificity and accuracy [14, 15]. CI for LR were calculated using the Log method [16, 17].

## Results

### Patients

**Fig. 1:**
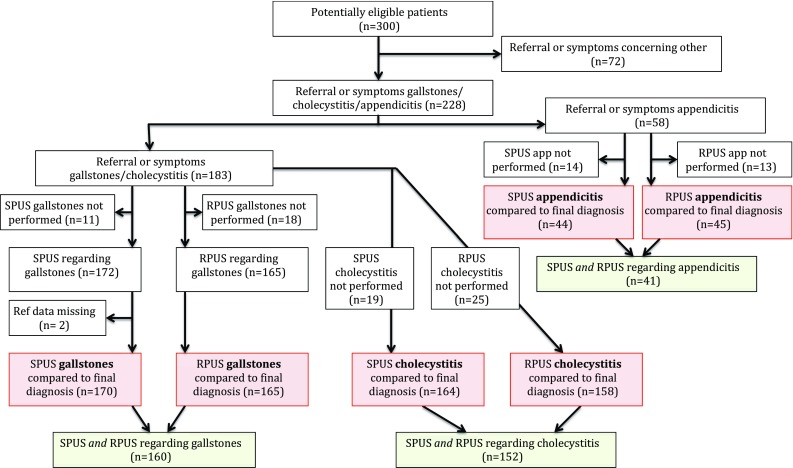
Flowchart included patients

Of the 300 eligible patients, 228 met the criteria for suspected biliary disease (*n* = 183) and/or appendicitis (*n* = 58) and were included for further analysis (Fig. 1). Baseline characteristics for the two groups are shown in Tables 1 and 2, respectively.

**Table 1:** Patient characteristics biliary disease (total *n* = 183)

Patient characteristics	*N* (%)	Mean/median (range)
Sex		
Male	80 (43.7)	
Female	103 (56.3)	
Age (years)		54^a^ (19–92)
Height (meters)		1.69^a^ (1.45–2.01)
Weight (kg)		75.9^a^ (40–125)
BMI (kg/m^2^)		26.3^a^ (13.8–47.5)
Admitted	114 (62.3)	
Way of referral*		
ED	88 (48.1)	
Surgery Dpt**	61 (33.3)	
Other	25 (13.7)	
RUQ pain	96 (52.5)	
RUQ tenderness	63 (34.4)	
Gallstone-specific referral	145 (79.2)	
WBC		8.8^b^ (2.2–26.1)
CRP		11^b^ (1–554)

*N* total number of patients, *BMI* body mass index, *ED* emergency department, *RUQ* right upper quadrant, *WBC* white blood cell count, *CRP* C-reactive protein

^a^Mean

^b^Median

*Information not available in 9 patients

**Surgery ward (58) outpatient clinic (3)

**Table 2:** Patient characteristics appendicitis (total *n* = 58)

Patient characteristics	*N* (%)	Mean/median (range)
Sex		
Male	22 (37.9)	
Female	36 (62.1)	
Age (years)		34.7^a^ (18–89)
Height (meters)		1.72^a^ (1.50–2.01)
Weight (kg)		71.0^a^ (47–106)
BMI (kg/m^2^)		23.9^a^ (19.1–31.0)
Admitted	35 (60.3)	
Way of referral*		
ED	35 (60.3)	
Surgery Dpt**	18 (31.0)	
Other	4 (6.9)	
RLQ pain	45 (77.6)	
RLQ tenderness	39 (67.2)	
Appendicitis-specific referral	46 (79.3)	
WBC		9.0^b^ (4.1–16.1)
CRP		16.5^b^ (1–275)

*N* total number of patients, *BMI* body mass index, *ED* emergency department, *RLQ* right lower quadrant, *WBC* white blood cell count, *CRP* C-reactive protein

^a^Mean

^b^Median

*Information not available in 1 patient

**Surgery ward

### Biliary disease

Among the 183 patients, with suspected biliary disease, 74 patients were shown to have gallstones and 21 had acute cholecystitis. Final diagnoses for all 183 patients are shown in Table 3.

**Table 3:** Final diagnoses, suspected biliary disease

Final diagnosis	Number (%)
Cholangitis	2 (1.1)
Cholecystitis	21 (11.5)
Choledocholithiasis	3 (1.6)
Cholelithiasis	29 (15.8)
Dyspepsia	1 (0.5)
Gastroenteritis	1 (0.5)
Hepatitis	1 (0.5)
Ileus	1 (0.5)
Malignancy	12 (6.6)
NSAP	68 (37.2)
Pancreatitis	19 (10.4)
Peptic ulcer	3 (1.6)
Pyelonephritis	1 (0.5)
UTI	1 (0.5)
Other	20 (10.9)
Total	183 (100)

*NSAP* non-specific abdominal pain, *UTI* urinary tract infection

#### Gallstones

**Fig. 2:**
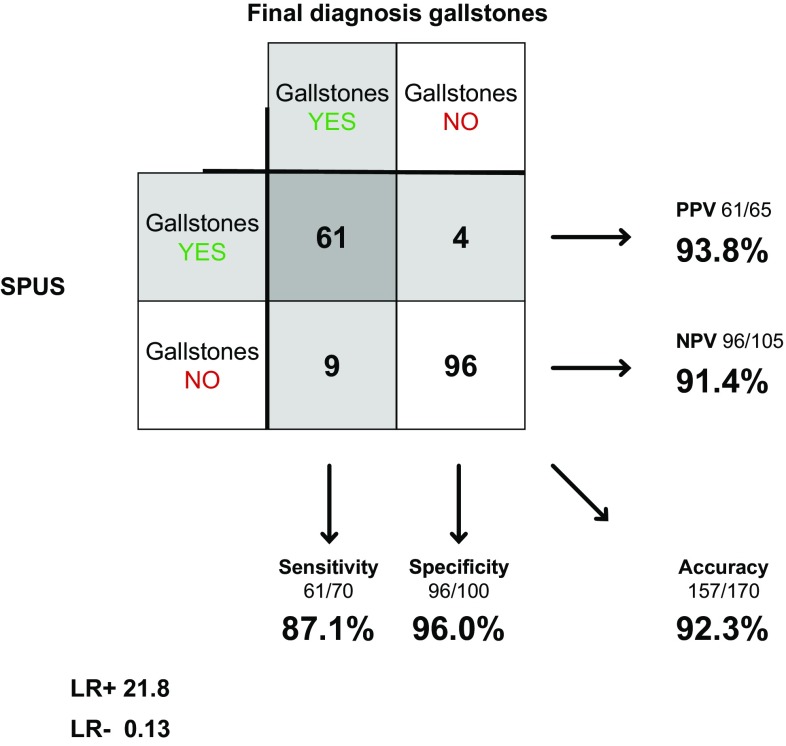
SPUS in diagnosing gallstones

Surgeons examined 172 of the 183 patients in concerns of gallstones, including 70 of the 74 patients with confirmed gallstones. Reference standard (final diagnosis) was missing in two cases (no radiology or operation was performed), leaving 170 comparable cases. Sensitivity was 87.1% (95% CI, 77.3–93.1%), specificity 96.0% (90.1–98.4%) and accuracy 92.3% (87.4–95.5%). Positive likelihood ratio (LR+) was 21.8 (8.30–57.2), and negative likelihood ratio (LR−) 0.13 (0.07–0.25) (Fig. 2).

**Fig. 3:**
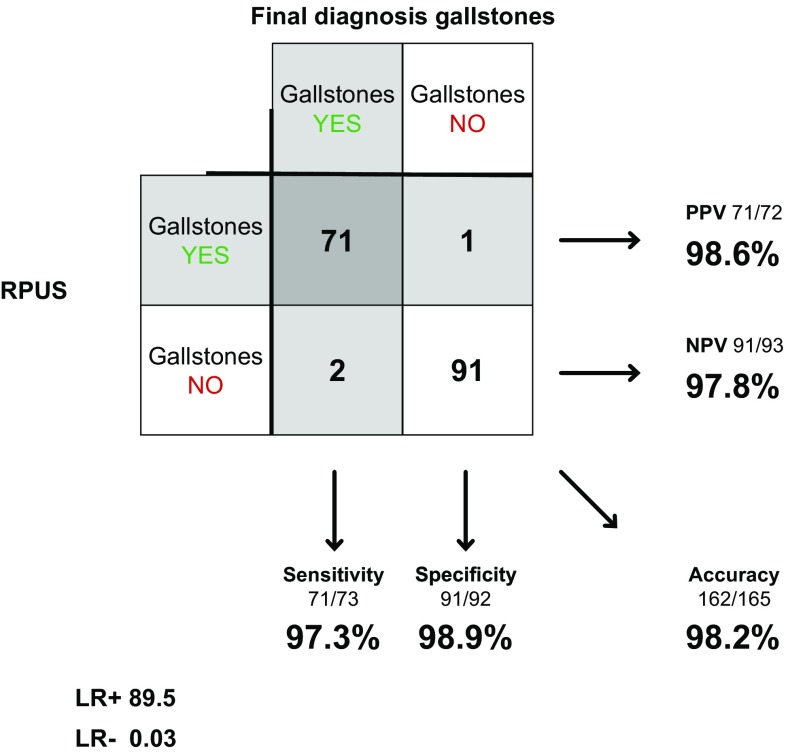
RPUS in diagnosing gallstones

Radiologists examined 165 of the 183 patients in concerns of gallstones, including 73 of the 74 confirmed cases. Sensitivity was 97.3% (90.6–99.3%), specificity 98.9% (94.1–99.8%) and accuracy 98.2% (94.8–99.4%). LR+ was 89.5 (12.7–629) and LR− 0.03 (0.01–0.11) (Fig. 3).

One hundred and sixty of the patients were examined both by surgeon and radiologist. Inter-observer agreement (Cohen’s kappa) between surgeon and radiologist regarding gallstones was 0.79 (good agreement). There was no systematic difference between surgeons and radiologists in how often the diagnosis was set (*p* = 0.454).

#### Cholecystitis

**Fig. 4:**
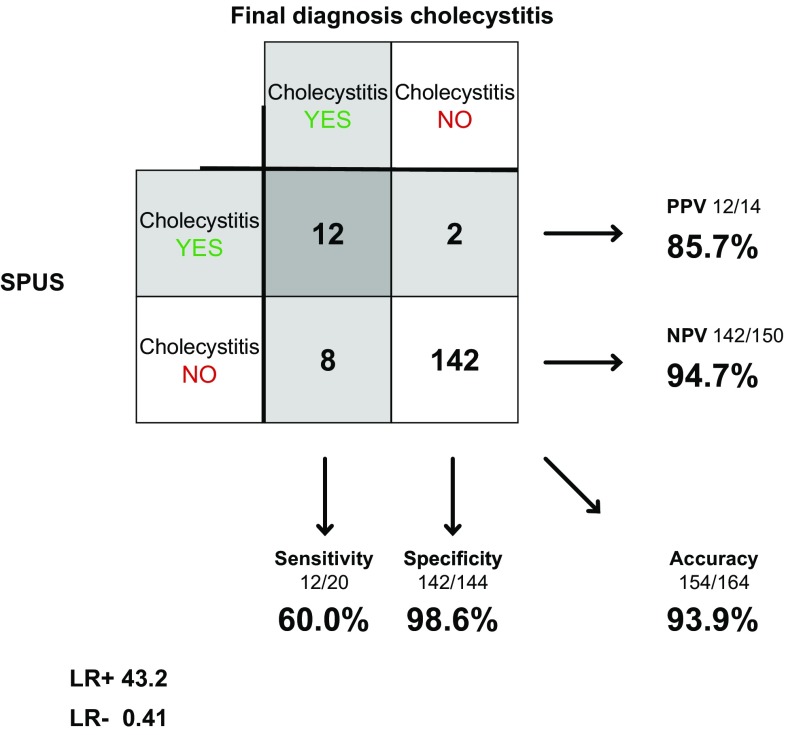
SPUS in diagnosing acute cholecystitis

Surgeons examined 165 of the 183 patients in concerns of cholecystitis. In one patient, the surgeon couldn’t find the gallbladder, leaving 164 examinations to be compared to final diagnosis. Sensitivity was 60.0% (38.7–78.1%), specificity 98.6% (95.1–99.6%) and accuracy 93.9% (89.1–96.7%). LR+ was 43.2 (10.4–179) and LR− 0.41 (0.24–0.69) (Fig. 4).

**Fig. 5:**
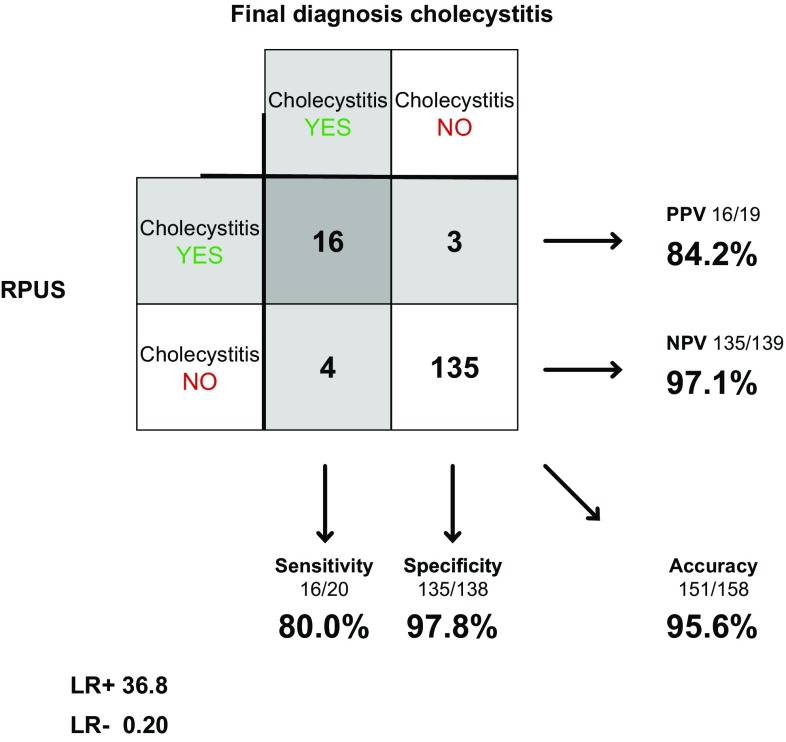
RPUS in diagnosing acute cholecystitis

Radiologists examined 158 of the 183 patients in concerns of cholecystitis. Sensitivity was 80.0% (56.3–94.3%), specificity 97.8% (93.8–99.3%) and accuracy 95.6% (91.1–97.8%). LR+ was 36.8 (11.8–115) and LR− 0.20 (0.09–0.49) (Fig. 5).

One hundred and fifty-two of the patients were examined both by surgeon and radiologist. Cohen’s kappa regarding cholecystitis was 0.61 (good agreement). There was no systematic difference between surgeons and radiologists in how often the diagnosis was set (*p* = 0.227).

#### False negative cases

Ten patients with acute cholecystitis were missed either by the surgeon or the radiologist or both. Characteristics of the false negative cases are presented in Table 4. Radiologists found six cases, which the surgeons missed, while surgeons found two cases missed by the radiologists. Surgeons and radiologists agreed in negative finding in two cases. Four of the 10 patients had a white blood cell (WBC) count more than 10 (10^9^/L), and four had a C-reactive protein (CRP) more than 30 (mg/L). Two patients had a temperature higher than 37.5 °C. All of the patients presented either with pain or tenderness in the RUQ. Three of these 10 patients fulfilled the criteria of acute cholecystitis according to TG13 at the time of the scanning, but all of them fulfilled the criteria later during their hospital stay. Four of the patients were not fasting at the time of the scanning, and information about fasting was missing in one case. The rest of the patients had been fasting for at least 6 h. Four of the patients had BMI >30, of which two had BMI >35.

**Table 4:** False negative results regarding cholecystitis, SPUS (*n* = 8) and RPUS (*n* = 4)

Patient Number	SPUS Gallstones	RPUS Gallstones	SPUS Cholecystitis	RPUS Cholecystitis	WBC Count (10^9^/L)	CRP (mg/L)	RUQ Pain	RUQ Tenderness	Temp > 37.5	TG 13 Criteria	Acute Surgery	Histopathology
69	+	+	–	–	13.3	91	+	+	–	–	–	MD
97	+	+	–	+	9	3	+	+	+	+	–	MD
181	–	+*	–	+	7.2	23	+	+	–	–	–	MD
192	–	+	–	+	7	7	+	–	–	–	+	MD
205	+	+	–	+	9.2	42	–	+	–	+	+	+**
251	+	+	+	–	7.6	19	+	+	–	–	–	MD
252	+	+	–	–	11.6	4	+	+	–	–	+	+
253	+	+	+	–	18	37	+	+	–	–	+	+
284	+	+	–	+	14	302	+	MD	+	+	–	MD
288	–	+***	–	+	6	1	–	+	–	–	–	MD

*RUQ* Right upper quadrant, *TG 13* Tokyo Guidelines 2013, *MD* missing data

*Radiologist reports highly echogenic bile (= sludge)

**Histopathology shows chronic inflammation, but was classified as “acute on chronic” according to peroperative findings

***Radiologist reports possibly small stones or sludge in the neck of the gallbladder

#### False positive cases

Totally, four false positive cases were noted. SPUS and RPUS agreed in positive finding in one patient. That patient presented with neither pain, nor tenderness in the RUQ. Laboratories were elevated with WBC count of 14 and CRP 48, and both surgeon and radiologist noted a thickened wall of the gallbladder, suggesting acute cholecystitis. This patient did not fulfill the TG13 criteria, and the final diagnosis was choledocholithiasis. The other false positive case from SPUS was diagnosed at discharge with gallstones. This patient presented with pain and tenderness in the RUQ and had a “slightly thickened gallbladder wall” according to the radiologist. Laboratories were completely normal, and the patient did not fulfill the TG13 criteria for acute cholecystitis. RPUS had two more false positive cases, one with normal laboratories, finally discharged with gallstones. The other one had no gallstones on RPUS, but acute cholecystitis according to the same examiner. Laboratories were normal also in this case and neither of these cases fulfilled the TG13 criteria. Information about fasting was missing in all of the false positive cases. None of the false positive cases had BMI >30.

### Appendicitis

Among the 58 patients analyzed regarding appendicitis, there were 15 confirmed cases. Final diagnoses for all 58 patients are shown in Table 5.

**Table 5:** Final diagnoses, suspected appendicitis

Final diagnosis	Number (%)
Appendicitis	15 (25.9)
Cholecystitis	1 (17)
Diverticulitis	1 (17)
Gastroenteritis	3 (5.2)
Hydronephrosis	1 (1.7)
Ileus	2 (3.4)
Mesenteric lymphadenitis	2 (3.4)
Malignancy	1 (1.7)
NSAP	25 (43.1)
Ovarian cyst	1 (1.7)
Ureterolithiasis	2 (3.4)
Other	4 (6.9)
Total	58 (100)

*NSAP* non-specific abdominal pain

**Fig. 6:**
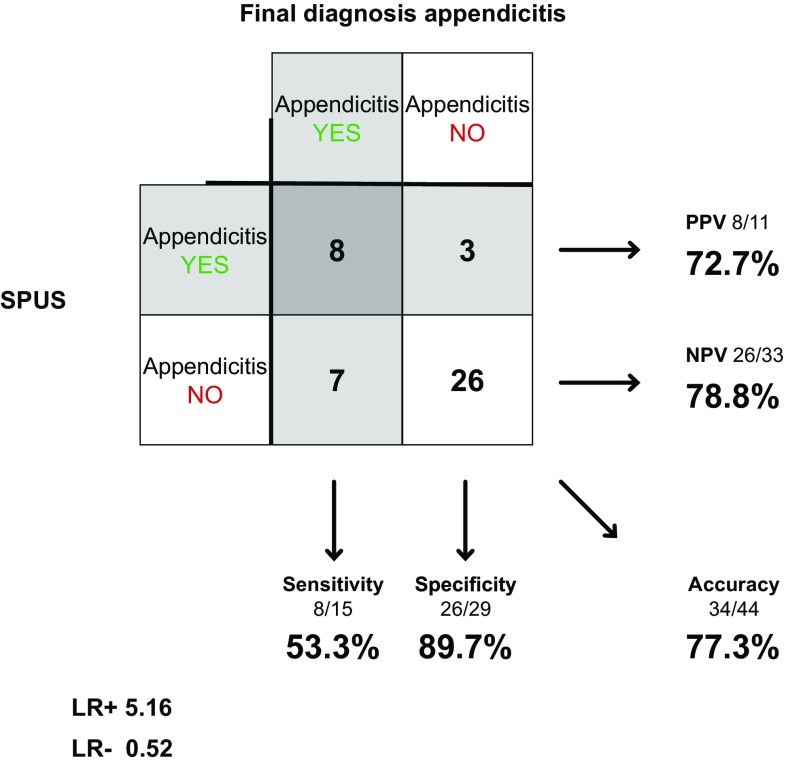
SPUS in diagnosing appendicitis

Surgeons examined 44 of the 58 patients in concerns of appendicitis. Sensitivity was 53.3% (30.1–75.2%), specificity 89.7% (73.6–96.4%) and accuracy 77.3% (63.0–87.2%). LR+ was 5.16 (1.60–16.6) and LR− 0.52 (0.30–0.91) (Fig. 6).

**Fig. 7:**
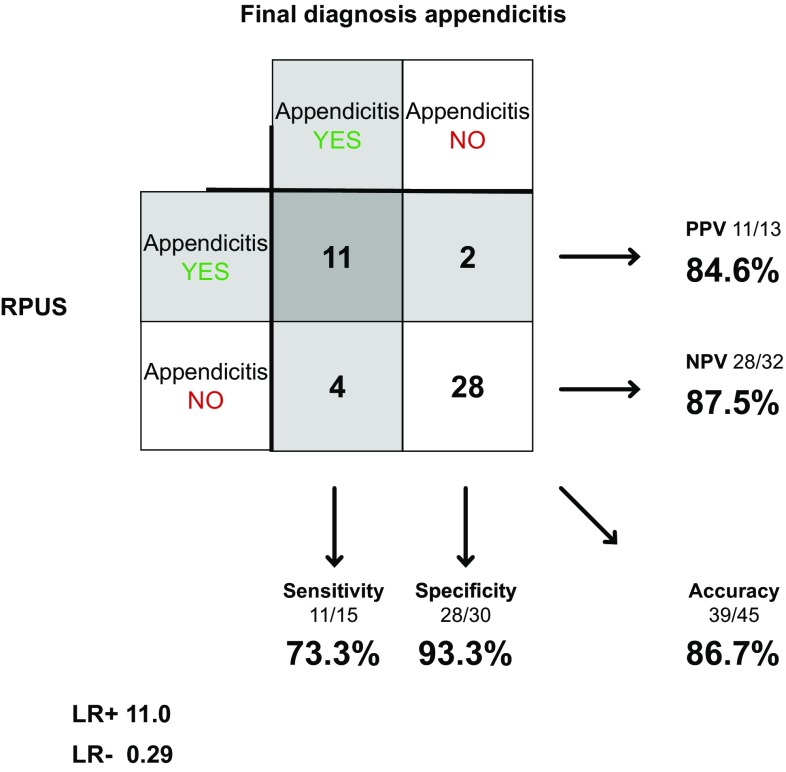
RPUS in diagnosing appendicitis

Radiologists examined 45 of the 58 patients in concerns of appendicitis. Sensitivity was 73.3% (48.1–89.1%), specificity 93.3% (78.7–98.2%) and accuracy 86.7% (73.8–93.7%). LR+ was 11.0 (2.79–43.4) and LR− 0.29 (0.12–0.66) (Fig. 7).

Forty-one of the patients were examined both by surgeon and radiologist. Cohen’s kappa regarding appendicitis was 0.41 (moderate agreement). There was no systematic difference between surgeons and radiologists in how often the diagnosis was set (*p* = 0.754).

#### False negative cases

In all seven cases missed by SPUS, the surgeon couldn’t find the appendix. RPUS correctly diagnosed five of these. RPUS missed four cases of appendicitis, of which SPUS correctly diagnosed two. All of these cases were confirmed with appendicitis at surgery. None of the false negative cases had a registered BMI >30. Information about BMI was missing in two of the cases.

#### False positive cases

SPUS misdiagnosed three and RPUS two cases as positive for appendicitis. SPUS and RPUS agreed in the positive finding in one of these cases, where examiners both found a tender 7-mm tubular structure in the RLQ. Three of the total four false positive cases were discharged with non-specific abdominal pain, and one with mesenteric lymphadenitis. None of the false positive cases had BMI >30.

## Discussion

Our results show that the diagnostic accuracy of SPUS concerning cholecystitis is considerably lower than for gallstones. The specificity, however, is still high, and the diagnostic value of the investigation is underlined by the high positive likelihood ratio. This holds for surgeons as well as radiologists. For appendicitis, both surgeons and radiologists reach a rather low sensitivity (53.3 vs. 73.3%). The high specificity and likelihood ratio though show that the examination still has a diagnostic value.

Hence, our study shows that it is more complicated to diagnose both cholecystitis and appendicitis with US, compared to gallstones. Although some studies, with exceptionally fine results for SPUS and RPUS regarding these diagnoses, have been presented [18], our results are well consistent with the reviewed literature and previous larger studies [9, 10]. In the systematic review by Carroll et al [19], in which it is concluded that SPUS can be regarded a sensitive and specific modality for the detection of appendicitis and gallstones, the included studies had results with higher sensitivity and specificity than in our study. However, in several of these studies the inclusion criteria were quite narrow and the prevalence of disease (appendicitis or gallstones) was considerably high, which might have affected the reported sensitivity and specificity [20]. As also mentioned by Carroll et al., one must consider observer bias in some of the included studies, since surgeons assessing the outcome were not blinded to the examinations, or in fact performed the US themselves.

Although this study focuses on the diagnostic accuracy of SPUS with respect to gallstones, cholecystitis and appendicitis, we also chose to compare the surgeon’s bedside examination (with a portable machine) to the radiologist’s examination (with a high end machine) for each diagnosis to get an approximation of the overall difficulty in examining these patients. RPUS is since many years accepted as the gold standard for diagnosing gallstones. It is also the recommended examination to confirm cholecystitis [13]. Limitations of the ultrasound examination for these diagnoses, but for patients with high BMI and non-fasting patients, are rarely discussed. We looked closer at the diagnosis of cholecystitis in patients who were misdiagnosed by SPUS and RPUS. It seems that early stage of the disease may contribute to a considerable amount of patients not fulfilling the diagnostic criteria of TG13 at the time of the scanning, as shown in Table 4, which might have affected the accuracy. BMI seems to be of some but limited importance for diagnostics in our material. Among cholecystitis cases, there were six individuals with BMI >30, of which four were misdiagnosed either by surgeon (three) or radiologist (two), one missed by both. The patient with highest BMI (39.7) was correctly diagnosed by SPUS but missed by RPUS. We found no systematic difference between SPUS and RPUS in how often the diagnosis was set, although the sensitivity (60.0 vs. 80.0%) differs quite a lot. However, it is hard to draw conclusions from this, considering the low prevalence of the disease in this cohort. In a wider aspect, if you look at SPUS as a piece of a diagnostic puzzle, alongside with other pieces such as auscultation, percussion, palpation and laboratory tests, sensitivity as low as 60% might be quite acceptable, especially when specificity and LR+ is high and the examination is without side effects. One could argue that perhaps the lower sensitivity for SPUS could be outweighed, to some extent, by the advantage of accessibility and owning the whole clinical picture.

RPUS for appendicitis is looked upon differently compared to acute cholecystitis. Most clinicians are aware of the problem with sensitivity and consider RPUS an adjunct to the clinical examination with the possibility to confirm but not exclude the diagnosis [8, 10]. Our results indicate that SPUS could be used in the same manner both for cholecystitis and appendicitis.

The included patients in our study represent a wide range of different diagnoses causing acute abdominal pain. The prevalence of each of the studied conditions well represents the normal range of differential diagnoses seen at the ED [3, 8, 21, 22], with appendicitis as a possible exception. The low prevalence of appendicitis in this study may be due to other preferred diagnostic imaging for appendicitis such as computed tomography chosen at our ED. The range of differential diagnoses, however, is one of the strengths of the study, making it clinically relevant and lowers the risk of selection bias.

Another strength with our work is that we have elucidated the accuracy of not only SPUS, but also RPUS in the study. This allows us to compare SPUS to standard care in the cohort. It also lets us draw general conclusions about US examinations for the diagnoses studied. The inclusion of gallstone diagnosis, although studied before, also strengthens the study. If not included, relative low accuracies for cholecystitis and appendicitis could be conferred to low US proficiency. The accuracies for RPUS and SPUS for the detection of gallstones now contradict such reasoning.

## Conclusion

SPUS is reliable in diagnosing gallstones. Diagnosing cholecystitis and appendicitis with US is more challenging for both surgeons and radiologists. We believe that SPUS could be used as an adjunct to the clinical examination with the possibility to confirm but not exclude these diagnoses. Further studies are needed to elucidate the difficulties with bedside US in cholecystitis and appendicitis.
